# Jejunal histopathology, metagenome, and mucosal transcriptome of broilers after an enteric challenge and fed diets with different fiber types and concentrations

**DOI:** 10.1016/j.psj.2026.107215

**Published:** 2026-06-02

**Authors:** R.W. Tabish, Y. Lin, S.J. Rochell, W.J. Pacheco, M.A. Bailey, W.A. Dozier, F.J. Hoerr, K. Robinson, R. Hauck

**Affiliations:** a Department of Poultry Science, Auburn University, Auburn, AL 36849, USA; b Veterinary Diagnostic Pathology, LLC, Polkton, NC 28135, USA; c USDA-ARS Poultry Research Unit, Mississippi State, MS 39762, USA; d Department of Pathobiology, Auburn University, Auburn, AL 36849, USA

**Keywords:** Dietary fiber, Enteric infection, Microbiome, Jejunal transcriptomics, Multi‑omics integration

## Abstract

This study investigated the efficacy of various dietary fiber sources and combinations in mitigating subclinical enteric infection in broilers. Using a randomized complete block design, 2,160 d-old YP x Ross 708 male broilers were assigned to eight treatments. These included an unchallenged control and a challenged control, followed by six dietary treatments applied to challenged broilers. The dietary treatments consisted of fiber supplementation with oat hulls (**OH**) or soy hulls (**SH**), either alone or in combination with wheat middlings (**WM**) or sugar beet pulp (**SBP**). Birds were challenged with *Eimeria* spp. followed by *Clostridium perfringens*, and a multi-omics approach was employed to analyze jejunal histopathology, microbiome, and host mucosal transcriptome. While the enteric challenge induced significant histopathological changes, fiber combinations including OH-WM and OH-SBP significantly (*P* < 0.05) reduced cumulative pathology scores. The challenge caused a shift toward *Lactobacillus crispatus* dominance in the microbiome. Each fiber source altered the microbiome distinctively: OH increased *Romboutsia* sp., OH-SBP enriched beneficial *Limosilactobacillus* spp., and SH combinations enhanced butyrate-producing *Dysosmobacter welbionis*. Transcriptome analysis revealed that fiber supplementation suppressed inflammatory pathways while upregulating cell cycle progression and DNA repair pathways. Integration of bacteriome with host gene expression data revealed coordinated associations, including a link between *Glutamicibacter protophormiae, Spirosoma, Eggerthella*, and *Blautia* through host genes APOB, DSEL, and ENPP7, indicating a correlation of fiber-degrading bacteria with host lipid metabolism and extracellular matrix remodeling. These findings suggest that combining insoluble and soluble fibers may create a more resilient gut environment against enteric challenges through complementary mechanisms, with OH based combinations notably exhibiting reduced pathology, stronger anti-inflammatory response and suppression of opportunistic species.

## Introduction

The removal of in-feed antibiotics and ionophore anticoccidials in raised without antibiotics or no antibiotics ever broiler production has resulted in increased problems with enteric pathogens, most notably the protozoa *Eimeria* spp. and the bacterium *Clostridium perfringens*, which act synergistically to cause necrotic enteritis (**NE**). *Eimeria* driven epithelial damage creates a nutrient rich environment in the gut that favors overgrowth of toxigenic *C. perfringens*, leading to clinical or subclinical NE, impaired nutrient absorption, and performance losses (Engster et al., 2002; Cervantes, 2015; Cervantes and McDougald, 2023). Challenge studies and field observations consistently show that diet composition and gut homeostasis are central determinants of NE development, with predisposing factors such as high dietary protein, non-starch polysaccharides, and disturbed intestinal microbiome increasing disease risk (Riddell and Kong, 1992; Kaldhusdal and Skjerve, 1996; Fernando et al., 2011; Emami et al., 2021).

Dietary fiber has been studied as one of the potential solutions to influence gut physiology and the microbiome during enteric challenge. Insoluble fiber sources, including oat hulls, stimulate gizzard development, lower gizzard pH, and modulate digesta transit, improving upstream digestive function and creating conditions less favorable for pathogen proliferation (Kheravii et al., 2018; Wróblewska et al., 2022; Akram et al., 2025). More fermentable fiber fractions and oligosaccharides like xylo-oligosaccharides and pectin selectively enrich beneficial taxa and support cross-feeding microbial networks that enhance production of short-chain fatty acids (**SCFAs**) linked to barrier support and colonocyte energy metabolism (Wils-Plotz et al., 2013; Davies et al., 2024; Castro et al., 2024). The SCFAs, including butyrate, can directly or indirectly inhibit *C. perfringens* growth and toxin production and attenuate excessive inflammatory signaling in the gut, providing biologically solution for diet-mediated NE mitigation (Timbermont et al., 2010; Gomez-Osorio et al., 2021).

While these effects are well supported, many broiler studies emphasize performance and lesion scores without addressing how fiber-driven microbial shifts simultaneously translate into metabolic functional changes and host transcriptomic responses, leaving important knowledge gaps. Integrative studies which connect microbial composition with their metabolic capacity and host transcriptional responses are needed to explain why particular fiber sources and combinations are protective under challenge. Here, we applied a multi-omics approach to test insoluble fibers, oat hulls (**OH**) and soy hulls (**SH**) alone or combined with more fermentable fiber sources, wheat middlings (**WM**) or sugar beet pulp (**SBP**) in birds challenged with *Eimeria* spp. and *C. perfringens*. By investigating the jejunal histopathology, bacteriome, mycobiome, and virome alongside profiling microbial metabolic functions and jejunal mucosal transcriptome, we identified fiber-specific molecular mechanisms that enrich beneficial microbial populations and align host transcriptional response toward epithelial renewal with controlled inflammation. These mechanistic insights provide an evidence-based rationale for precision use of fiber sources and combinations to support gut resilience during enteric challenge in antibiotic-free production.

## Materials and methods

### Experimental design, animals, and housing

All animal care and experimental procedures were approved by the Auburn University Institutional Animal Care and Use Committee (2023-5223). The study was conducted as a randomized complete block design, with pen location serving as the blocking factor. A total of 2,160-day-old YP × Ross 708 male broiler chicks were sourced from a commercial hatchery and randomly distributed among 8 dietary treatment groups, with each treatment replicated in 9 floor pens housing 30 birds each. Birds were housed in floor pens (2.79 m^2^) containing wood shaving bedding, and each pen was equipped with hanging feeders and a nipple drinker line, providing *ad libitum* access to feed and water throughout the 35-d study. Barn temperature was maintained at 33 °C at placement and gradually reduced to 20 °C by 32 days of age. The photoperiod was set to 23 h of light and 1 h of dark for the first 7 days, followed by 20 h of light and 4 h of dark for the remainder of the experiment, with light intensity managed at 30, 10, and 5 lux for days 1 to 7, 8 to 14, and 15 to 35, respectively.

### Diets

Corn soybean-meal based diets were formulated following the nutrient recommendations of primary breeding company (Aviagen, 2022) for YP x Ross 708 broilers. The experimental diets were designed to evaluate various fiber sources and concentrations. The dietary treatments consisted of an unchallenged control group (NC) and a challenged control group (PC), both fed a diet with no supplemental fiber, as well as six challenged groups fed diets containing either 3% OH, 3% SH, 1.5% OH and 1.5% WM (OH-WM), or 1.5% OH and 1.5% SBP (OH-SBP), 1.5% SH and 1.5% WM (SH-WM), or 1.5% SH and 1.5% SBP (SH-SBP). The analyzed fiber composition of the ingredients was as follows: OH contained 82.07% NDF (Neutral Detergent Fiber), 46.57% ADF (Acid Detergent Fiber), and 35.49% hemicellulose; SBP contained 35.69% NDF, 22.88% ADF, and 12.80% hemicellulose; SH contained 63.79% NDF, 46.67% ADF, and 17.12% hemicellulose; and WM contained 38.52% NDF, 12.20% ADF, and 26.32% hemicellulose. All fiber ingredients were included at the expense of corn. Experimental diets were manufactured at the Auburn University Feed Mill at the Charles Miller Research and Education Center. The fiber ingredients were obtained from a supplier and distributor of feed ingredients and agricultural byproducts (Cerco Group) and were selected because they are commonly used feed commodities readily available for practical application in commercial poultry feed formulations.

### Enteric challenge model

To induce an enteric challenge, birds in the challenged treatment groups were orally gavaged on d 14 with 1 mL of a commercial trivalent coccidial vaccine (Advent®, Huvepharma). The vaccine was administered at 10 times the manufacturer’s recommended dose to provide approximately 2,300 oocysts of *E. maxima* and a combined 9,600 oocysts of *E. acervulina* and *E. tenella* per bird. Birds in the NC group received 1 mL of sterile phosphate-buffered saline. Following a dietary transition from starter to grower feed on d 17, birds in the challenged groups were subsequently gavaged on d 18 with 1 mL of a brain heart infusion (**BHI**) broth culture containing a NetB-negative strain of *C. perfringens* (1 × 10⁸ colony-forming units/mL) (Gulizia et al., 2025). Birds in the NC group received a sham gavage of 1 mL of sterile BHI broth (Becton, Dickinson and Company, Sparks, MD).

### Sample collection

On d 21, i.e. 3 days post *C. perfringens* challenge, 8 birds per pen were euthanized via CO₂ asphyxiation followed by cervical dislocation in accordance with American Veterinary Medical Association guidelines (AVMA, 2020). For histological analysis, a 2 cm jejunal segment, located 5 cm proximal to Meckel’s diverticulum, was collected from 3 birds per pen and immediately fixed in 10% neutral buffered formalin until processing. For metagenomic analysis, approximately 2 g of jejunal content was collected from 1 bird per pen, immediately placed on ice, and subsequently stored at −80 °C. For transcriptome analysis, mucosal scrapings from 5 to 10 cm segments of the mid-jejunum were collected from one bird per pen, preserved in RNAlater (Thermo Fisher Scientific™, Waltham, MA), incubated at 4 °C for 24 h, after which the RNAlater was drained and the tissue stored at −80 °C until processing.

### Histological examination

Fixed jejunal tissues sampled at 21 d of age were routinely processed, embedded in paraffin, sectioned at 5 µm, and stained with hematoxylin and eosin (**H&E**). Three intact cross-sections from each sample were mounted per slide. Slides were digitally scanned at 20X magnification using a DP600 scanner (Ventana) and evaluated using Navify® Digital Pathology Software (Roche). For morphometric analysis, calibrated images of the entire intestinal circumference were captured and analyzed using Fiji software (ImageJ, National Institutes of Health). An observer, blinded to treatment allocations, measured villus height (**VH**) and crypt depth (**CD**) on 10 anatomically representative, well-oriented villus-crypt units per sample to calculate the **VH:CD** ratio.

Furthermore, a board-certified veterinary pathologist, blinded to treatment groups until data analysis, scored histopathological lesions according to the methods described by Kinstler et al. (2024) using a 0 to 5 scale (0 = normal; 1 = minimal; 2 = mild; 3 = moderate; 4 = marked; 5 = severe). Evaluated parameters included coccidiosis with a predominant presence of *Eimeria maxima* with rare *E. acervulina*, gut-associated lymphoid tissue (**GALT**) hyperplasia i.e. diffuse and focal lymphocytic expansion in lamina propria, dysbacteriosis, necrotic enteritis, cystic crypts, lamina propria heterophils, intraepithelial lymphocytes (**IEL**), increased goblet cells, increased mucus, misshaped villus tips, and congestion. From these individual parameters, a cumulative pathology score, i.e. sum of all lesions excluding coccidiosis, and a cumulative immune response score, i.e. sum of GALT and IEL scores, were calculated for each sample.

### Metagenomic analysis

Genomic DNA was extracted from approximately 200 mg of jejunal content using the QIAamp DNA Stool Kit (Qiagen, Hilden, Germany) following the manufacturer’s protocol. DNA concentration and purity were confirmed using an Implen NanoPhotometer® N60 (Implen GmbH, Munich, Germany). Shotgun metagenomic sequencing was performed by Azenta Life Sciences (South Plainfield, NJ). Libraries were sequenced on an Illumina platform using a 2 × 150 bp paired-end configuration, intended to generate approximately 10 Gb of data per sample with a quality range of ≥80% of bases exceeding a Phred quality score of Q30. Raw sequence data were deposited in the NCBI Sequence Read Archive (Accession: PRJNA1187240).

Sequencing data quality was assessed with FastQC (Andrews, 2010) and low-quality reads and adapters were trimmed using Trimmomatic v0.39 (Bolger et al., 2014). The resulting high-quality reads were then processed to remove host-derived sequences using HISAT2 v2.2.0 (Kim et al., 2019b) with the chicken reference genome GRCg7b, retaining only non-host reads for downstream metagenomic analysis. These host-removed paired-end reads were taxonomically classified using Kraken2 (Wood et al., 2019) with a comprehensive BLAST core nucleotide database (core_nt), using a stringent criterion for taxon mapping requiring at least 20% of a reads constituent k-mers to map to a single taxon, thus minimizing ambiguous or low-confidence taxonomic assignments. Species-level abundance estimation was performed using Bracken (Lu et al., 2017) with a Bayesian statistical model specifically set at a read length of 150 bp. Functional profiling was conducted using HUMAnN v3.6 (Beghini et al., 2021) on interleaved paired-end reads, with MetaPhlAn used for taxonomic profiling during the HUMAnN workflow.

### Transcriptomic analysis

Total RNA was extracted from approximately 15 mg of jejunal mucosal tissue utilizing the RNeasy Mini Kit (Qiagen, Hilden, Germany), incorporating both bead mill homogenization and on-column DNase treatment. The concentration and purity of RNA were evaluated using an Implen NanoPhotometer® N60 (Implen GmbH, Munich, Germany), with A260/280 ratios of ≥1.8 considered acceptable. RNA integrity assessment was conducted on an Agilent 5400 Fragment Analyzer (Agilent Technologies, Ramsey, MN), preferring samples with RNA Integrity Numbers ≥ 4.0 for subsequent library preparation. RNA sequencing, including poly-A enrichment, was outsourced to Novogene Corporation Inc. (Sacramento, CA) and performed on an Illumina NovaSeq X Plus platform to generate 150 bp paired-end reads with ∼30 million read pairs per sample. The sequencing data were deposited in National Center for Biotechnology Information Sequence Read Archive under accession number PRJNA1187003.

For bioinformatic analysis, FastQC was used for quality assessment of raw reads, followed by adapter trimming using Trimmomatic v0.39. The resulting clean reads were mapped to the *Gallus gallus* reference genome (**GRCg7b**) using HISAT2. Gene-level quantification was performed with HTSeq-count v1.13.3 (Anders et al., 2015). Differential gene expression was performed in R (v4.5.0) (R Core Team, 2023), using PC as the reference group and comparing all challenged fiber supplemented treatments against it. Additionally, NC group was used as the reference to assess the effect of the challenge (PC vs. NC). For each comparison, the dataset was filtered to include only genes with cumulative counts ≥20 across all relevant samples. Differentially expressed genes (**DEGs**) were identified using DESeq2 (Love et al., 2014), applying threshold criteria of adjusted *P*-value < 0.05 and absolute log₂ fold change > 1. For pathway enrichment analysis, genes were preranked according to metric sign (log_2_ (FoldChange)) × (-log_10_ (*P*-value)) and analyzed using the Preranked module in GSEA (Gene Set Enrichment Analysis) software (Subramanian et al., 2005). Hallmark pathways with false discovery rate (**FDR**) q-values ≤ 0.05 were considered significantly enriched and are presented with their normalized enrichment scores (**NES**).

### Bacteriome and host gene correlation analysis

To explore the relationship between the bacteriome and host gene expression, overlapping samples from both datasets were identified and analyzed using R following the methods described by Priya et al. (2022). Species-level microbial abundances were normalized via Centered Log-Ratio (**CLR**) transformation (Nearing et al., 2022), while host gene counts underwent variance stabilizing transformation (**VST**). The overall similarity between the normalized bacteriome and host gene datasets was evaluated using Procrustes analysis. To uncover groups of microbes and genes that exhibit correlated patterns, Sparse Canonical Correlation Analysis (**sCCA**) was applied to the standardized data. For pinpointing specific microbial taxa linked to individual host genes, gene-wise Lasso regression was performed, modeling each gene’s VST-normalized expression as a function of CLR-transformed microbial abundances. Statistically significant associations were subsequently filtered and visualized as a network depicting host-microbe interactions.

### Statistical analysis

All statistical analyses were conducted using R. Each treatment was represented by 9 replicate pens in a randomized complete block design. The pen was considered the experimental unit for jejunal histomorphometry and histopathology, whereas the individual bird (each bird representing one pen) was the experimental unit for metagenomic and transcriptome analyses.

For jejunal histopathology, histomorphometry, and microbial alpha-diversity data were analyzed using the Kruskal-Wallis test due to non-normal distribution, followed by Dunn’s post-hoc test with Bonferroni correction when significant differences (*P* ≤ 0.05) were detected. Prior analysis confirmed no significant block effects on these parameters, supporting the use of a simplified statistical model. Microbial beta-diversity was analyzed using permutational multivariate analysis of variance (**PERMANOVA**) with the adonis function in the vegan package (Oksanen et al., 2025). Microbial differential abundance analysis data were analyzed using DESeq2, the PC group was used as reference for all pairwise comparisons except PC vs. NC, where NC was the baseline group to access the effect of challenge. Taxa were considered significantly differentially abundant at an adjusted *P*-value (*P_adj_*) threshold of <0.05 based on the Benjamini-Hochberg method, and results are reported with corresponding log_2_ fold changes (Log_2_FC). For metabolic functional pathway analysis, HUMAnN3 generated pathway abundance data were filtered to exclude unmapped and unintegrated reads, as well as stratified pathways. Differential abundance analysis on these filtered data was then performed as described above, with significance defined at *P* < 0.05. Statistical methods specific to host gene expression are detailed in its respective sections above.

## Results

### Jejunal histology

**Fig. 1:**
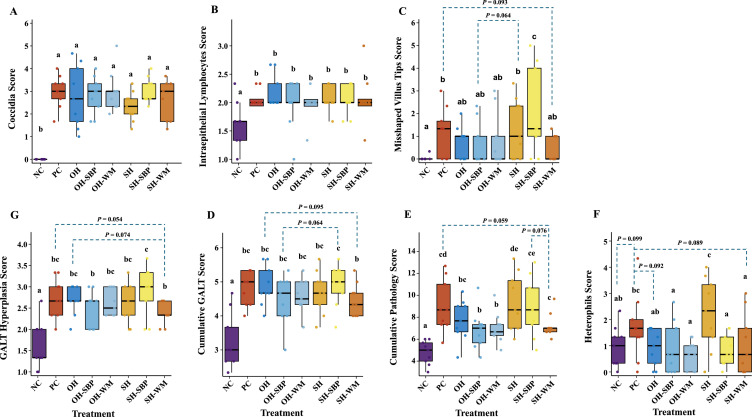
Histopathological scores in the jejunum of broilers across all treatment groups. The box plots display scores for: (A) coccidia, (B) intraepithelial lymphocytes (C) misshapen villus tips, (C) GALT hyperplasia (D) cumulative GALT (E) cumulative pathology and (F) heterophils. The horizontal line within each box represents the median, box boundaries represent the interquartile range, and whiskers extend to 1.5 times the interquartile range, with individual data points overlaid. Superscripts denote significant differences between treatment groups as determined by a post-hoc test (*P* < 0.05); groups not sharing a common letter are significantly different. Marginal significance is denoted with exact P value. Treatment groups are as follows: NC, Negative Control (unchallenged); PC, Positive Control (challenged); and challenged groups receiving diets containing OH, Oat Hulls; SH, Soy Hulls; OH-SBP, Oat Hulls with Sugar Beet Pulp; OH-WM, Oat Hulls with Wheat Middlings; SH-SBP, Soy Hulls with Sugar Beet Pulp; and SH-WM, Soy Hulls with Wheat Middlings.

**Fig. 2:**
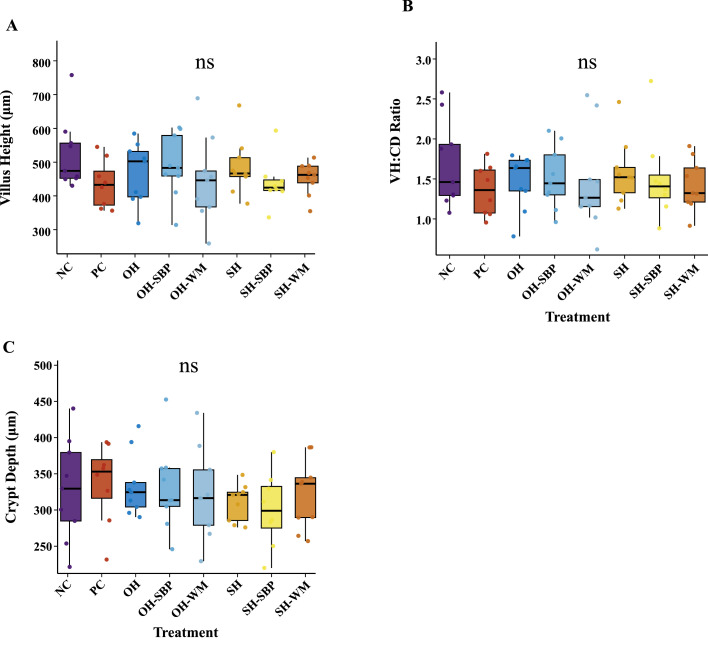
Jejunal histomorphology of broilers across all treatment groups. The box plots display key morphological measurements from the jejunum: (A) villus height (µm), (B) VH:CD ratio, and (C) crypt depth (µm). The horizontal line within each box represents the median, box boundaries represent the interquartile range, and whiskers extend to 1.5 times the interquartile range, with individual data points overlaid. Difference among treatment groups were not significant based on the Kruskal-Wallis test (*P* > 0.05). Treatment groups are as follows: NC, Negative Control (unchallenged); PC, Positive Control (challenged); and challenged groups receiving diets containing OH, Oat Hulls; SH, Soy Hulls; OH-SBP, Oat Hulls with Sugar Beet Pulp; OH-WM, Oat Hulls with Wheat Middlings; SH-SBP, Soy Hulls with Sugar Beet Pulp; and SH-WM, Soy Hulls with Wheat Middlings.

The enteric challenge significantly affected jejunal histopathology with challenged birds having higher coccidia scores (*P* < 0.001), intraepithelial lymphocytic infiltration (*P* < 0.05), misshaped villus tips (*P* < 0.001), and GALT hyperplasia (*P* < 0.001), leading to significantly elevated cumulative GALT and pathology scores (*P* < 0.001) compared to unchallenged birds (Fig. 1). While histomorphometric parameters were not significantly altered, challenged birds had numerically reduced VH and VH:CD ratio (Fig. 2).

Coccidia lesion scores did not differ (*P* > 0.05) among challenged and fiber-supplemented groups, although the group receiving only SH in feed exhibited slightly lower numerical values (Fig. 1). Intraepithelial lymphocytic infiltration was also similar (*P* > 0.05) among fiber-supplemented groups and the PC, with groups receiving OH-WM and SH-WM showing marginally lower means. Misshaped villus tips were more prevalent in the group receiving SH-SBP supplemented diet (*P* < 0.05), whereas overall OH-supplemented treatments had relatively lower means (*P* > 0.05). GALT hyperplasia scores approached significance (*P* = 0.054), in the SH-WM group exhibiting the lower score compared to the PC. Among the challenged groups, SH-SBP showed a numerical increase in GALT hyperplasia, while OH-SBP had lower scores; the remaining treatments were comparable to the PC (*P* > 0.05). Cumulative GALT scores were numerically highest in SH-SBP and lowest in SH-WM among challenged groups. However, cumulative pathology scores were significantly reduced in groups supplemented with OH-WM, OH-SBP (*P* < 0.05), and SH-WM (*P* = 0.059) compared with the PC. Heterophil infiltration was markedly higher in SH compared with other challenged, fiber-supplemented groups (*P* < 0.05), whereas birds receiving both soluble and insoluble fiber combination in feed had significantly lower infiltration compared with the PC (*P* < 0.05). No differences (*P* > 0.05) in VH, CD, or VH:CD ratio were detected among challenged groups (Fig. 2), although VH tended to be lower in SH-SBP and PC, and highest in NC and OH-SBP. The highest VH:CD ratio was observed in NC, followed by OH-SBP among challenged groups.

### Jejunal bacteriome

**Fig. 3:**
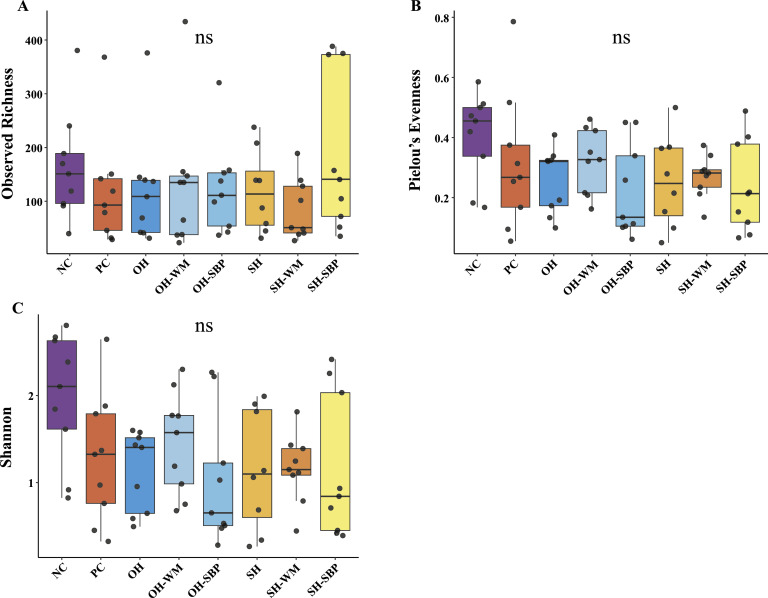
Effect of different dietary fiber sources on jejunal microbial alpha diversity. Box plots represent three alpha diversity metrics: (A) observed features (richness), (B) Pielou’s evenness, and (C) the Shannon diversity index. In each plot, the horizontal line indicates the median, box boundaries represent the interquartile range, and whiskers extend to 1.5 times the interquartile range. Individual data points are overlaid. Difference among treatment groups were not significant based on the Kruskal-Wallis test. Treatment groups are as follows: NC, Negative Control (unchallenged); PC, Positive Control (challenged); and challenged groups receiving diets containing OH, Oat Hulls; SH, Soy Hulls; OH-SBP, Oat Hulls with Sugar Beet Pulp; OH-WM, Oat Hulls with Wheat Middlings; SH-SBP, Soy Hulls with Sugar Beet Pulp; and SH-WM, Soy Hulls with Wheat Middlings.

**Fig. 4:**
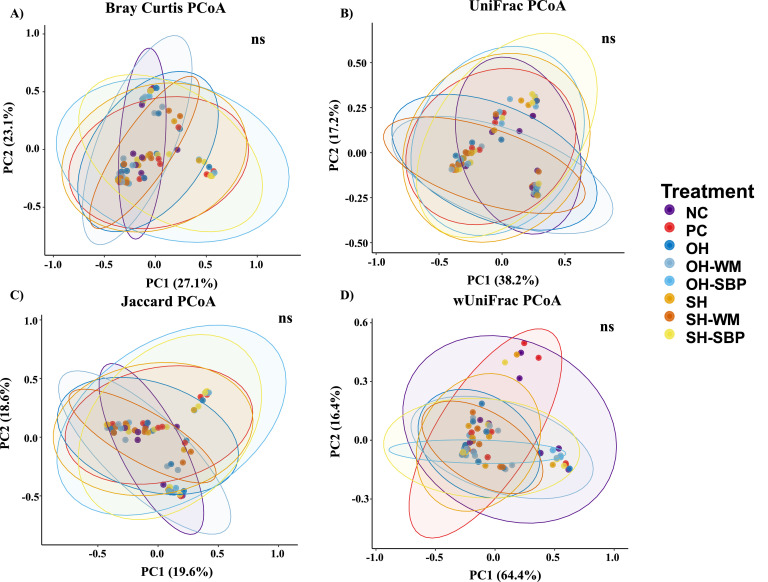
Beta diversity of jejunal microbial communities in response to dietary fiber supplementation. Principal Coordinate Analysis (PCoA) plots were generated based on four distance metrics: (A) Bray-Curtis dissimilarity, (B) unweighted UniFrac distance, (C) Jaccard dissimilarity, and (D) weighted UniFrac distance. Each point represents the microbial community of an individual sample, colored by its respective treatment group. Ellipses represent the 95% confidence interval for the centroid of each group. The percentage of variation explained by each principal coordinate axis is indicated in brackets. Difference among treatment groups were not significant based on the PERMANOVA. Treatment groups are as follows: NC, Negative Control (unchallenged); PC, Positive Control (challenged); and challenged groups receiving diets containing OH, Oat Hulls; SH, Soy Hulls; OH-SBP, Oat Hulls with Sugar Beet Pulp; OH-WM, Oat Hulls with Wheat Middlings; SH-SBP, Soy Hulls with Sugar Beet Pulp; and SH-WM, Soy Hulls with Wheat Middlings.

**Fig. 5:**
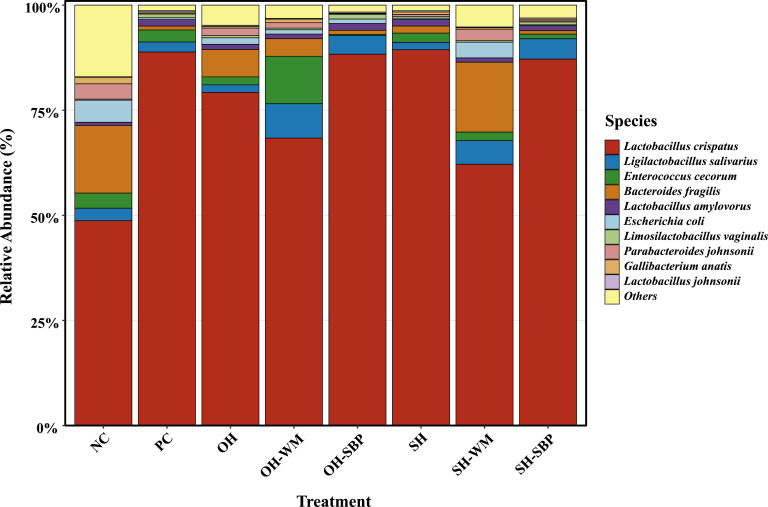
Relative abundance of the top 10 bacterial species in Jejunal samples across all treatment groups. This stacked bar plot illustrates the mean relative abundance of the most prevalent bacteria at the species level. Each bar represents a distinct treatment group, and the colored segments correspond to the relative abundance of the top 10 identified species. The ‘Others’ category comprises all remaining, less abundant species not ranked in the top 10. Treatment groups are as follows: NC, Negative Control (unchallenged); PC, Positive Control (challenged); and challenged groups receiving diets containing OH, Oat Hulls; SH, Soy Hulls; OH-SBP, Oat Hulls with Sugar Beet Pulp; OH-WM, Oat Hulls with Wheat Middlings; SH-SBP, Soy Hulls with Sugar Beet Pulp; and SH-WM, Soy Hulls with Wheat Middlings.

Shotgun metagenomic sequencing of jejunal samples generated a total of 2.52 billion reads, with an average of 35.5 million reads per sample (Figure S1). Alpha and beta diversity analysis revealed no significant (*P* > 0.05) differences in jejunal bacteriome composition among treatment groups (Fig. 3, Fig. 4). The relative abundance of the top 10 most dominant bacterial species in the jejunum across treatment groups is shown in Fig. 5. In the unchallenged control group, *Lactobacillus crispatus* was the predominant species (48.72%), followed by *Bacteroides fragilis* (16.05%), *Escherichia coli* (5.27%), *Parabacteroides johnsonii* (3.62%), and *Enterococcus cecorum* (3.61%). The enteric challenge shifted the bacterial profile with *L. crispatus* becoming more dominant (88.85%) compared to NC. Concurrently, there were reductions in *B. fragilis* (0.94%), *E. coli* (0.45%), and *P. johnsonii* (0.24%).

**Fig. 6:**
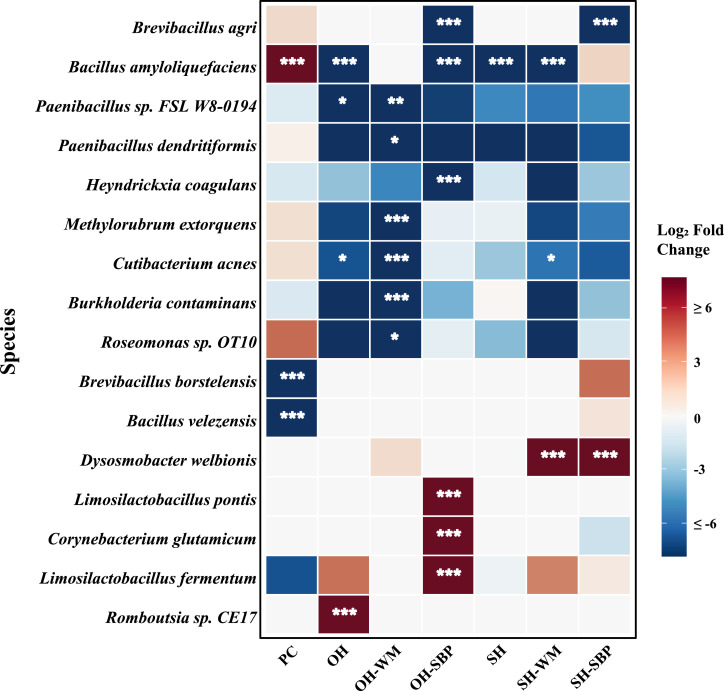
Differentially abundant bacterial taxa in jejunal contents across dietary treatment groups. The heatmap displays the Log₂ fold change of species identified as significantly different in at least one comparison (*P*_*adj*_ < 0.05). Rows correspond to individual bacterial species, and columns represent pairwise comparisons of each treatment group. Colors indicate the magnitude of fold change relative to the PC, with red shades denoting higher relative abundance and blue shades denoting lower relative abundance. For the first comparison (PC vs. NC), the NC serves as the reference, and the direction of regulation reflects changes in the PC. Asterisks within the cells represent the level of statistical significance for that specific comparison: *P_*adj*_< 0.05, **P_*adj*_ < 0.01, and ***P_*adj*_< 0.001. Treatment groups are as follows: NC, Negative Control (unchallenged); PC, Positive Control (challenged); and challenged groups receiving diets containing OH, Oat Hulls; SH, Soy Hulls; OH-WM, Oat Hulls with Wheat Middlings; OH-SBP, Oat Hulls with Sugar Beet Pulp; SH-WM, Soy Hulls with Wheat Middlings; and SH-SBP, Soy Hulls with Sugar Beet Pulp.

Based on differential abundance analysis results, the enteric challenge significantly (*P_adj_* < 0.001) increased *Bacillus amyloliquefaciens* (log_2_FC = 21.89) while decreasing *Bacillus velezensis* (log_2_FC = −23.57) and *Brevibacillus borstelensis* (log_2_FC = −23.21) in PC compared to NC (Fig. 6). The supplementation of OH in the feed of the challenged group increased *Romboutsia sp.* CE17 (log_2_FC = 23.35) compared to PC, while decreasing *B. amyloliquefaciens* (log_2_FC = −22.22), *Paenibacillus sp.* FSL W8-0194 (log_2_FC = −6.44, *P_adj_* = 0.03), and *Cutibacterium acnes* (log_2_FC = −5.18, *P_adj_* = 0.02). The treatment receiving OH, along with WM, decreased the relative abundance of six bacterial species compared to PC, including *Burkholderia contaminans* (log_2_FC = −27.82), *Methylorubrum extorquens* (log_2_FC = −26.97), *Paenibacillus dendritiformis* (log_2_FC = −8.51, *P_adj_* = 0.03), *Roseomonas sp.* OT10 (log_2_FC = −9.05, *P_adj_* = 0.03), *Paenibacillus sp.* FSL W8-0194 (log_2_FC = −7.22, *P_adj_* = 0.006), and *Cutibacterium acnes* (log_2_FC = −6.79). The OH, along with SBP supplementation, decreased the relative abundance of *Heyndrickxia coagulans* (log_2_FC = −25.43), *Brevibacillus agri* (log_2_FC = −24.55), and *B. amyloliquefaciens* (log_2_FC = −23.21) compared to PC, while increasing *Limosilactobacillus pontis* (log_2_FC = 25.75), *Limosilactobacillus fermentum* (log_2_FC = 21.39), and *Corynebacterium glutamicum* (log_2_FC = 20.99). The supplementation of SH in the challenged group only decreased *B. amyloliquefaciens* (log_2_FC = −21.16) compared to PC. The combination of SH and WM supplementation in the challenged group decreased *B. amyloliquefaciens* (log_2_FC = −21.48) and *C. acnes* (log_2_FC = −4.46, *P_adj_* = 0.021), while increasing *D. welbionis* (log_2_FC = 23.13). Finally, the SH, along with SBP supplementation, increased the relative abundance of *Dysosmobacter welbionis* (log_2_FC = 25.79) while decreasing *Brevibacillus agri* (log_2_FC = −24.82) compared to PC.

### Jejunal mycobiome

**Fig. 7:**
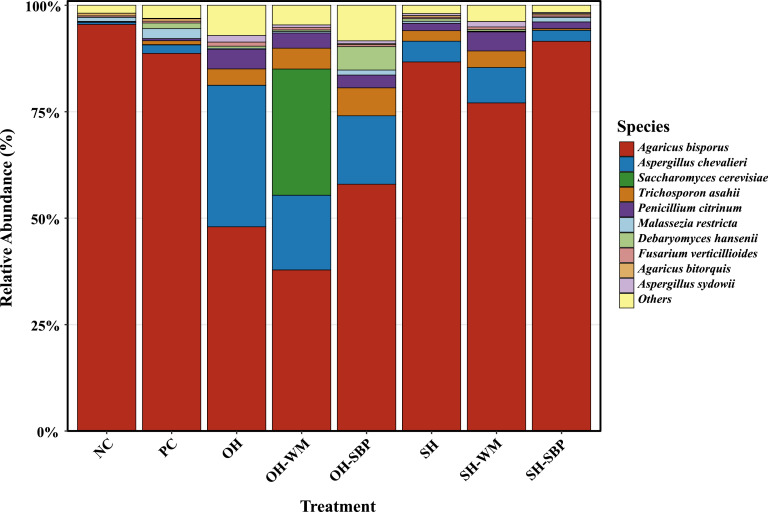
Relative abundance of the top 10 fungal species in jejunal samples across all treatment groups. This stacked bar plot illustrates the mean relative abundance of the most prevalent fungi at the species level. Each bar represents a distinct treatment group, and the colored segments correspond to the relative abundance of the top 10 identified fungal species. The ‘Others’ category comprises all remaining, less abundant species not ranked in the top 10. Treatment groups are as follows: NC, Negative Control (unchallenged); PC, Positive Control (challenged); and challenged groups receiving diets containing OH, Oat Hulls; SH, Soy Hulls; OH-SBP, Oat Hulls with Sugar Beet Pulp; OH-WM, Oat Hulls with Wheat Middlings; SH-SBP, Soy Hulls with Sugar Beet Pulp; and SH-WM, Soy Hulls with Wheat Middlings.

Alpha and beta diversity analyses of the jejunal mycobiome revealed no significant differences among treatment groups, consistent with observations from the bacteriome. The relative abundances of the 10 most dominant fungal species across treatment groups are presented in Fig. 7. In the unchallenged control group, *Agaricus bisporus* dominated the community (95.51%), followed by *Malassezia restricta* (0.95%), *Agaricus bitorquis* (0.64%), and *Fusarium verticillioides* (0.30%). The enteric challenge shifted the fungal profile, with *A. bisporus* declining to 88.70%, while *M. restricta* (2.35%), *Aspergillus chevalieri* (1.99%), *Debaryomyces hansenii* (1.32%), and *Trichosporon asahii* (0.90%) increased. Oat hull supplementation further reduced *A. bisporus* (47.96%) while increasing *A. chevalieri* (33.23%). The OH-WM treatment showed the lowest proportion of *A. bisporus* (37.84%) and was uniquely enriched in *Saccharomyces cerevisiae* (29.64%). OH-SBP maintained intermediate levels of *A. bisporus* (57.97%) with higher *D. hansenii* (5.50%). In contrast, SH-based treatments generally sustained higher proportions of *A. bisporus* (SH: 86.69%, SH-WM: 77.07%, SH-SBP: 91.55%) compared to oat hull-based diets. Notably, the enterically challenged PC group exhibited a significantly higher relative abundance of *Aspergillus flavus* (log_2_FC ≈ 23, *P_adj_* < 0.001) compared to both the unchallenged control and all fiber-supplemented groups.

### Eimeria abundance

Analysis of jejunal apicomplexan read counts showed that the enteric challenge significantly increased *Eimeria maxima* abundance in PC compared with NC (*P* = 0.011). Similar increases were observed for *Eimeria tenella* (*P* = 0.073) and *Eimeria acervulina* (*P* = 0.333), although these did not reach statistical significance (Table 1). Comparisons of fiber treatments against PC revealed no significant differences in *Eimeria* counts for any of the three major species.

**Table 1:** Bracken-corrected raw read counts from Kraken2 taxonomic assignments across Jejunal samples of broilers provided diets with different dietary fiber sources and concentrations on d 21.

Apicomplexan species	Bracken-adjusted raw read counts based on Kraken2 classifications^1^							
	NC^2^	PC	OH	OH-WM	OH-SBP	SH	SH-WM	SH-SBP
*Eimeria maxima*	92611	2518466	2025579	4671261	2972879	3796361	1457021	1053843
*Eimeria acervulina*	14218	31598	58471	386953	53052	21461	15908	196384
*Eimeria tenella*	3594	12925	6130	12302	15124	6504	10224	6192
*Eimeria praecox*	0	2410	4357	11674	1667	3107	1579	4312
*Eimeria necatrix*	31	198	112	132	96	145	81	101
*Eimeria mitis*	10	53	25	735	69	66	11	18
*Cyclospora cayetanensis*	0	130	36	92	63	94	8	42
*Plasmodium gallinaceum*	30	0	0	0	0	5	20	0

1 Raw read counts represent the number of sequencing reads classified by Kraken2 at the species level using a confidence threshold of 0.2, requiring at least 20% of k-mers to support a taxonomic assignment, and subsequently corrected for abundance estimation using Bracken.

2 Treatment groups are as follows: NC, Negative Control (unchallenged); PC, Positive Control (challenged); and challenged groups receiving diets containing OH, Oat Hulls; SH, Soy Hulls; OH-SBP, Oat Hulls with Sugar Beet Pulp; OH-WM, Oat Hulls with Wheat Middlings; SH-SBP, Soy Hulls with Sugar Beet Pulp; and SH-WM, Soy Hulls with Wheat Middlings.

In addition to these predominant species, DNA of other apicomplexans were detected in the jejunum, including *Eimeria mitis, Eimeria necatrix*, and *Eimeria praecox. Cyclospora cayetanensis* was consistently detected across all challenged groups but was present only at minimal levels in NC. Trace reads corresponding to *Plasmodium gallinaceum* were also observed in some treatments.

### Jejunal virome

As this analysis was based on shotgun DNA sequencing, the characterization is limited to DNA viruses. At the taxonomic level, the jejunal DNA virome was predominantly composed of bacteriophages across all groups. *Caudoviricetes* sp. was the most abundant taxon, accounting for approximately 70-90% of the relative abundance across all treatment groups (Figure S2). The community structure in the PC appeared to show a modest shift, with a lower abundance of *Caudoviricetes* sp. and a corresponding increase in the relative abundance of *Phaecoctavirus* sp. No viral species showed significant differential abundance across treatment groups (*P_adj_* > 0.05).

Additionally, very few archaeal reads were detected in the jejunal samples, preventing meaningful comparative analysis of this domain.

### Microbial functional pathways

**Fig. 8:**
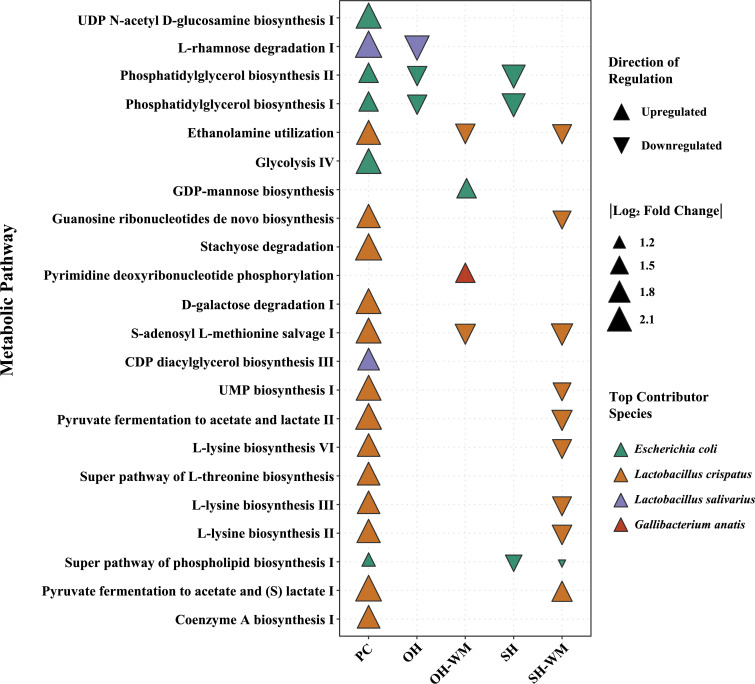
Differentially abundant metabolic pathways in jejunal microbial communities. This plot displays metabolic pathways identified using HUMAnN2 that were significantly different (*P* < 0.05) relative to the positive control (PC) and were relevant to the study. The y-axis lists the metabolic pathways, while the x-axis represents pairwise comparisons among treatment groups. Triangle size is proportional to the absolute log₂ fold change, indicating the magnitude of the change in pathway abundance. The color of each triangle denotes the primary bacterial species contributing to the observed differential abundance. The direction of each triangle corresponds to the regulation of the pathway, where upward-pointing triangles indicate upregulation and downward-pointing triangles indicate downregulation. All comparisons use the PC as the reference, except for the first comparison (PC vs. NC), where the NC serves as the reference, and the direction of regulation reflects changes in the PC. Treatment groups include: NC, Negative Control (unchallenged); PC, Positive Control (challenged); OH, Oat Hulls; SH, Soy Hulls; OH-WM, Oat Hulls with Wheat Middlings; SH-WM, Soy Hulls with Wheat Middlings.

The enteric challenge markedly altered the functional potential of the jejunal microbiota. Compared to unchallenged birds, challenged birds exhibited several increased pathways essential for microbial growth and metabolism (Fig. 8). Notably, nucleotide biosynthesis pathways, including UMP biosynthesis I (log_2_FC = 2.18, *P* = 0.004) and guanosine ribonucleotides de novo biosynthesis (log_2_FC = 1.99, *P* = 0.005), were enriched, primarily driven by *Lactobacillus crispatus*, which dominated the jejunal microbiota post-challenge. Similarly, the super pathway of L-threonine biosynthesis (log₂FC = 1.69, *P* = 0.035) was elevated, also largely attributed to *L. crispatus*. Other significantly upregulated pathways included UDP-N-acetyl-D-glucosamine biosynthesis I (log₂FC = 2.71, *P* = 0.027) and glycolysis IV (log₂FC = 2.74, *P* = 0.032), predominantly driven by *Escherichia coli*.

Fiber supplementation changed the microbial functional pathways when compared to PC. The OH treatment decreased pathways involved in bacterial membrane synthesis, such as phosphatidylglycerol biosynthesis I and II (log_2_FC = −1.39, *P* = 0.019; log_2_FC = −1.39, *P* = 0.019), mainly driven by *E. coli*. Combining OH and WM in challenged birds increased pathways for pyrimidine deoxyribonucleotide phosphorylation (log_2_FC = 1.53, *P* = 0.033), attributed to *Gallibacterium anatis*, and GDP-mannose biosynthesis (log_2_FC = 1.45, *P* = 0.038), driven by *E. coli*. Conversely, SH supplementation decreased the super pathway of phospholipid biosynthesis (log_2_FC = −1.41, *P* = 0.013), primarily due to *E. coli*. SH combined with WM further downregulated key metabolic pathways, including S-adenosyl-l-methionine salvage I (log_2_FC = −1.80, *P* = 0.027) and guanosine ribonucleotides de novo biosynthesis (log_2_FC = −1.51, *P* = 0.034), largely driven by *L. crispatus*.

### Jejunal mucosal transcriptome

**Fig. 9:**
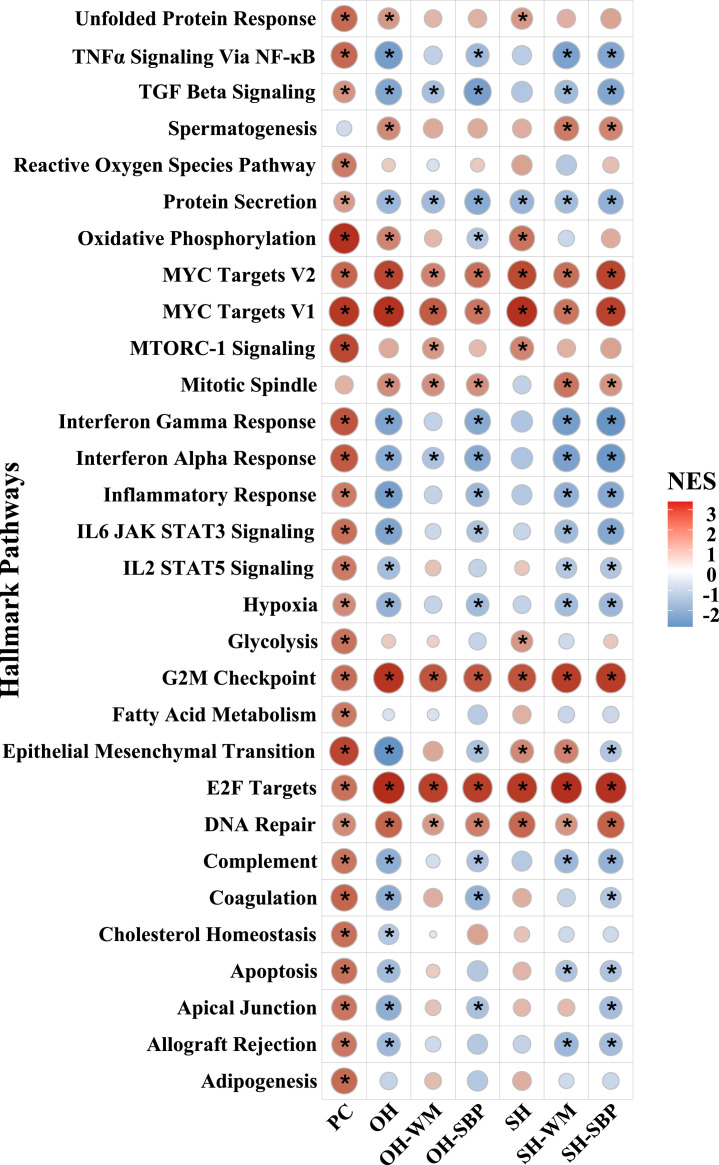
Gene Set Enrichment Analysis (GSEA) of Hallmark pathways in host jejunal tissue. This bubble heatmap displays the top 30 highly enriched pathways from GSEA based on the absolute NES. The y-axis lists the Hallmark gene sets, and the x-axis represents the comparisons of the treatment groups. The color of each bubble corresponds to the direction of enrichment, where red indicates pathways upregulated and blue indicates pathways downregulated relative to the PC, except for the first comparison (PC vs. NC), in which the NC serves as the reference and the direction of regulation reflects changes in the PC. The size of the bubble is proportional to the absolute value of the NES. Asterisks (*) denote a significant enrichment with a FDR q-value < 0.05. Treatment groups are as follows: NC, Negative Control (unchallenged); PC, Positive Control (challenged); and challenged groups receiving diets containing OH, Oat Hulls; SH, Soy Hulls; OH-WM, Oat Hulls with Wheat Middlings; OH-SBP, Oat Hulls with Sugar Beet Pulp; SH-WM, Soy Hulls with Wheat Middlings; and SH-SBP, Soy Hulls with Sugar Beet Pulp.

RNA sequencing of jejunal samples yielded an average of 25.9 million reads per sample. The enteric challenge induced significant changes in the jejunal transcriptome (Figure S3). There were 1,803 differentially expressed genes (*P_adj_* < 0.05) between the challenged and the unchallenged groups (Table 2). Of these, 720 DEGs were upregulated and 1,083 were downregulated in the PC group. Gene Set Enrichment Analysis with Hallmark gene sets (Fig. 9) showed that highly enriched pathways (FDR < 0.01) included oxidative phosphorylation (NES = +3.15), proto-oncogenes (**MYC**) targets V1 and V2 (NES = +3.03; NES = +2.32), and epithelial-mesenchymal transition (NES = +2.85). A strong inflammatory response was also present, with enrichment of the interferon-gamma response (NES = +2.58), interferon-alpha response (NES = +2.50), and tumor necrosis factor alpha (**TNFα**) signaling via nuclear factor kappa B (**NF-κB**) (NES = +2.28) pathways.

**Table 2:** Differentially expressed genes (DEGs) in the jejunal mucosa of broilers provided diets with different dietary fiber sources on d 21.

Comparison^1^	DEGs^2^	Upregulated^3^	Downregulated^4^
PC vs NC	1803	720	1083
OH vs PC	9	1	8
OH-WM vs PC	2	1	1
OH-SBP vs PC	1	1	0
SH vs PC	7	1	6
SH-WM vs PC	2	1	1
SH-SBP vs PC	1	1	0

1 All treatments except NC were challenged with Eimeria spp. on d 14 and 10⁸ CFU of C. perfringens on d 18, and samples were collected at d 21. Treatment groups are as follows: NC, Negative Control (unchallenged); PC, Positive Control (challenged); and challenged groups receiving diets containing OH, Oat Hulls; SH, Soy Hulls; OH-SBP, Oat Hulls with Sugar Beet Pulp; OH-WM, Oat Hulls with Wheat Middlings; SH-SBP, Soy Hulls with Sugar Beet Pulp; and SH-WM, Soy Hulls with Wheat Middlings.

2 Differentially expressed genes (DEGs) have been filtered based on P_adj_ < 0.05 and LogFC > ±1.

3 Upregulated genes have a log fold change of greater than +1 (LogFC > +1).

4 Downregulated genes have a log fold change of less than −1 (LogFC < −1).

Supplementation with dietary fiber altered the transcriptomic response to the enteric challenge. Compared to the challenged control, the inclusion of OH resulted in the suppression (FDR < 0.01) of pathways for the epithelial-mesenchymal transition (NES = −2.76), TNFα signaling via NF-κB (NES = −2.57), inflammatory response (NES = −2.47), and both interferon-gamma (NES = −2.34) and interferon-alpha (NES = −2.16) responses. Concurrently, the OH supplementation upregulated pathways involved in cell cycle progression, such as transcription factor (**E2F**) targets (NES = +3.39), G-phase to M-phase (**G2M**) checkpoint (NES = +3.08), and MYC targets V1 and V2 (NES = +3.12; NES = +2.83), along with DNA repair (NES = +2.37). The addition of OH with WM resulted in the enrichment of pathways related to cell cycle progression, including E2F targets (NES = +2.95), G2M checkpoint (NES = +2.62), and MYC targets V1 and V2 (NES = +2.49; NES = +1.89). A suppression of the interferon-alpha response (NES = −1.55, FDR = 0.044) and transforming growth factor (**TGF**) beta signaling (NES = −1.63, FDR = 0.031) was also observed. The combination of OH and SBP led to the upregulation of E2F targets (NES = +2.93), G2M checkpoint (NES = +2.61), and MYC targets V1 and V2 (NES = +2.11; NES = +2.18). This combination also suppressed pathways for TGF-beta signaling (NES = −2.46), the interferon-alpha (NES = −2.20) and interferon-gamma (NES = −2.19) responses, and TNFα signaling via NF-kB (NES = −1.78).

The inclusion of SH caused the enrichment (FDR < 0.01) of pathways for MYC targets V1 and V2 (NES = +3.14; NES = +2.76), E2F targets (NES = +3.00), G2M checkpoint (NES = +2.58), DNA repair (NES = +2.30), and oxidative phosphorylation (NES = +2.16). The only significantly suppressed pathway was protein secretion (NES = −1.88, FDR = 0.0018). The combination of SH and WM resulted in the downregulation of a broad range of immune pathways, including the interferon-gamma (NES = −2.49) and interferon-alpha (NES = −2.41) responses, and TNFα signaling via NF-kB (NES = −2.39). In parallel, it led to the upregulation of pathways for E2F targets (NES = +3.22), G2M checkpoint (NES = +2.98), and MYC targets V1 and V2 (NES = +2.10; NES = +2.19). Finally, the combination of SH and SBP resulted in suppressing key immune pathways, including the interferon-gamma (NES = −2.65) and interferon-alpha (NES = −2.63) responses, TNFα signaling via NF-kB (NES = −2.29), and IL-6 (Interleukin-6)/JAK (Janus Kinase)-STAT3 (Signal Transducer and Activator of Transcription 3) signaling (NES = −2.28). Simultaneously, it also caused the upregulation of pathways for E2F targets (NES = +3.21), G2M checkpoint (NES = +2.99), MYC targets V1 and V2 (NES = +2.89; NES = +2.83), and DNA repair (NES = +2.41).

### Correlation between bacteriome and host response

**Fig. 10:**
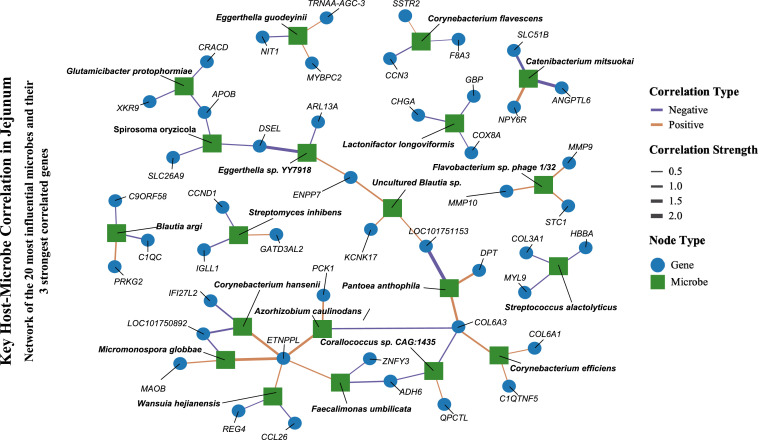
Network of the 20 most influential bacterial hubs and their primary host gene associations. This network visualizes the key interactions in the broiler jejunal bacteriome. The 20 bacteria with the strongest individual association coefficients in the dataset were first identified as “hubs” (green squares). For each of these bacterial hubs, lines (edges) connect to the three host genes (blue circles) with which they have the strongest positive (red edges) or negative (blue edges) association. The width of each edge is proportional to the absolute magnitude of the LASSO coefficient, indicating the strength of the interaction.

A statistically significant global association was established between the jejunal bacteriome and host gene expression (Procrustes analysis, correlation = 0.3781, *P* = 0.001), leading to the identification of a core functional module of bacteria and genes with strong co-variation (sCCA, correlation = 0.6073). A gene-by-gene Lasso regression model then identified specific host-microbe interactions (Fig. 10).

Network analysis revealed a distinct structural organization of host-microbe interactions, characterized by well-defined hubs and linear connection patterns. A prominent central hub was centered on the host gene ETNPPL, a key regulator of phospholipid metabolism. This node exhibited strong correlations with a taxonomically diverse group of microbes, including *Azorhizobium caulinodans, Corynebacterium hansenii, Micromonospora globbae, Wansuia hejianensis*, and *Faecalimonas umbilicata*. In addition to this hub, a major linear network of interconnected nodes extended across the graph. This pathway linked several fiber degrading microbial taxa through intermediate host genes related to lipid metabolism and extracellular matrix remodeling, beginning with *Glutamicibacter protophormiae* and connecting through nodes associated with *Spirosoma oryzicola, Eggerthella* sp., *Blautia* sp., and others. A second hub, organized around the host gene COL6A3, revealed both positive correlations with microbes such as *Pantoea anthophila* and negative correlations with others. The association between *Corynebacterium efficiens* and collagen genes COL6A1 and COL6A3 is especially relevant as *C. efficiens* produces propionate from fiber and can enhance collagen gene expression in intestinal tissues. Beyond these major hubs, the network exhibited distinct patterns of microbial associations. Certain taxa, such as *Flavobacterium* sp., were exclusively connected to host genes through positive correlations, suggesting potentially synergistic relationships. In contrast, microbes including *Streptococcus alactolyticus* and *Lactonifactor longoviformis* displayed uniformly negative correlations, indicative of possible antagonistic or inhibitory roles within the jejunal environment.

## Discussion

The present study investigated the efficacy of different dietary fiber sources, concentrations, and their combinations in mitigating the adverse effects of subclinical enteric infection with *Eimeria spp.* and *Clostridium perfringens* in broilers. The findings reveal complex interactions between dietary fiber supplementation, gut microbiome composition, and host transcriptomic responses during enteric challenge, providing insights into the mechanisms by which specific fiber sources may confer protection against intestinal pathogens.

The experimental challenge with *Eimeria* spp*.* followed by *C. perfringens* successfully induced subclinical enteric infection, as evidenced by significant histopathological changes in the jejunum. Challenged birds exhibited elevated coccidia scores, increased intraepithelial lymphocytic infiltration, misshaped villus tips, and GALT hyperplasia compared to unchallenged control. This histological profile aligns with previous reports of experimental NE models, where *Eimeria* infection predisposes birds to *C. perfringens* colonization through intestinal barrier disruption and mucin release (Zhao et al., 2022; Akerele et al., 2022; Goo et al., 2024). The numerical reduction in villus height and VH:CD ratio, though not statistically significant, suggests compromised absorptive capacity which has been associated with performance losses in birds with subclinical enteric infections (Awad et al., 2009).

Despite significant histopathological alterations, the enteric challenge did not significantly modify jejunal microbial alpha and beta diversity. However, it induced a taxonomic shift toward *Lactobacillus crispatus* dominance, reaching 88.85% in PC compared with 48.72% in NC, accompanied by concurrent reductions in *Bacteroides fragilis, Escherichia coli*, and *Parabacteroides johnsonii*. This microbial shift is consistent with observations by Lacey et al. (2018), who reported significant microbiota alterations during *C. perfringens* challenge. The dominance of *L. crispatus* in the challenged birds is interesting and noteworthy, as this species has been associated with enhanced intestinal barrier function and pathogen exclusion in poultry (Stanley et al., 2014; Kim et al., 2015; Zyl et al., 2019; Wu et al., 2024). The significant increase in *Bacillus amyloliquefaciens* in challenged birds is important too, as certain strains of this species have been reported to exhibit antimicrobial and immunomodulatory properties (Gharib-Naseri et al., 2020). However, it’s also possible that these proliferations may only indicate opportunistic colonization following disruption of the normal microbiota. The enteric challenge also altered the jejunal mycobiome, decreasing *Agaricus bisporus* dominance while increasing opportunistic fungi such as *Malassezia restricta, Aspergillus chevalieri*, and notably *Aspergillus flavus*. This is particularly interesting as *A. flavus* produces aflatoxins that can exacerbate intestinal inflammation and damage (Hernández-Ramírez et al., 2021). The observed fungal shifts represent an understudied aspect of NE pathogenesis, as the mycobiome’s role in poultry gut health has received limited attention compared to the bacterial microbiome (Yang et al., 2021).

In the host tissue, the challenge induced substantial transcriptomic changes, resulting in 1,803 differentially expressed genes, with significant enrichment of pathways related to oxidative phosphorylation, MYC targets, and epithelial-mesenchymal transition. The upregulation of interferon responses, including IFN-γ and IFN-α, along with TNFα signaling via NF-κB, indicates a robust pro-inflammatory response consistent with previous studies on host responses to *Eimeria* and *C. perfringens* (Zhou et al., 2009; Sarson et al., 2009). Such activation represents the classical Th1-type defense against intracellular protozoa like *Eimeria*, with IFN-γ serving as a signature cytokine mediating Th1 immune responses in avian coccidial infections (Lillehoj and Trout, 1996; Kim et al., 2019a; Gaghan et al., 2022). The enrichment of the epithelial-mesenchymal transition (EMT) pathway may further indicate ongoing tissue remodeling in response to epithelial damage, consistent with observations by Pham et al. (2021) regarding repair mechanisms during intestinal infection. However, this inflammatory signature not only reflects the host’s attempt to clear the infection but may also contribute to tissue damage and performance losses (Lu et al., 2020).

Both OH and SH treatments reduced the abundance of *B. amyloliquefaciens* compared to the PC, suggesting these insoluble fibers may restrict proliferation of certain opportunistic bacteria during infection. However, OH supplementation uniquely increased *Romboutsia sp.*, a genus associated with gastrointestinal health in multiple species (Song et al., 2024). This effect may contribute to the improved histopathological outcomes observed in OH-supplemented groups. The most striking difference between OH and SH treatments was observed in the mycobiome composition. Oat hulls supplementation substantially reduced *A. bisporus* dominance, with 47.96% in OH compared with 88.70% in PC, while increasing *A. chevalieri*. In contrast, SH maintained high proportions of *A. bisporus* at 86.69%. The biological significance of these mycobiome differences remains unclear, as the functional implications of different fungal communities in poultry are not well characterized. However, the significant reduction in *A. flavus* in all fiber-supplemented groups compared to PC suggests a protective effect against potentially toxigenic fungi, possibly through competitive exclusion or modified gut environment. At the transcriptomic level, OH supplementation was particularly effective at suppressing inflammatory pathways, including TNFα signaling via NF-κB, and interferon responses, while upregulating pathways involved in cell cycle progression and DNA repair. This dual action suggests OH may limit inflammatory damage while promoting epithelial regeneration. Similar immunomodulatory effects of insoluble fiber have been reported by Zeitz et al. (2019), who observed reduced pro-inflammatory cytokine expression in chickens fed diets containing insoluble fiber during *C. perfringens* challenge. SH supplementation alone resulted in higher heterophil infiltration compared to other challenged, fiber-supplemented groups, suggesting it may induce a stronger acute inflammatory response. However, SH also upregulated pathways related to cell cycle progression, DNA repair, and oxidative phosphorylation, indicating that despite the inflammatory response, epithelial renewal mechanisms were enhanced. Studies have shown that the insoluble fiber supplementation can support the higher VH and VH:CD (Zhang et al., 2023; Karimipour et al., 2025).

The combination of insoluble fibers with soluble fiber sources, either WM or SBP, yielded the most promising results in terms of histopathological improvement. Groups receiving OH-WM, OH-SBP, and SH-WM had significantly reduced cumulative pathology scores compared to PC. These combinations also resulted in significantly lower heterophil infiltration compared to PC, suggesting attenuated acute inflammation. These findings are consistent with Jha and Mishra (2021), who reviewed that soluble and insoluble fiber could improve intestinal health in broilers challenged with enteric infections. The fiber combinations had distinct effects on the microbiome. OH-WM reduced six bacterial species compared to PC, including potential pathogens like *Burkholderia contaminans* and opportunistic bacteria such as *Cutibacterium acnes*. OH-SBP uniquely increased beneficial *Limosilactobacillus* species including *L. pontis* and *L. fermentum*, which have been associated with improved intestinal barrier function and reduced inflammation in poultry (Guo et al., 2023). SH-WM and SH-SBP increased *Dysosmobacter welbionis*, a recently described butyrate-producing bacterium that may contribute to intestinal health through SCFA production (Roy et al., 2022). At the host tissue level, all fiber combinations downregulated inflammatory pathways while upregulating cell cycle progression. However, OH combinations appeared more effective at suppressing epithelial-mesenchymal transition, a process associated with intestinal fibrosis during chronic inflammation (Scharl et al., 2015). The SH-SBP combination was particularly effective at suppressing interferon responses and IL-6/JAK/STAT3 signaling, pathways that, while essential for pathogen clearance, can cause tissue damage when chronically activated (Matsuda, 2023). The microbial functional pathways analysis revealed that fiber supplementation altered microbial metabolic activities in ways that may benefit the host. For instance, OH-WM increased pathways for pyrimidine deoxyribonucleotide phosphorylation and GDP-mannose biosynthesis, potentially enhancing cellular proliferation and glycoprotein synthesis needed for mucosal repair (Löffler et al., 2005; Zhang et al., 2024). Conversely, SH-WM downregulated S-adenosyl-L-methionine salvage and guanosine ribonucleotides de novo biosynthesis, possibly limiting resources required for pathogen proliferation (Samant et al., 2008; North et al., 2020).

Integration of microbiome and transcriptome data revealed coordinated associations between specific jejunal microbes and host genes, rather than isolated effects. ETNPPL, a regulator of phospholipid metabolism, formed a central hub connected with *Azorhizobium caulinodans, Wansuia hejianensis, Micromonospora globbae*, and *Faecalimonas umbilicata*, all of which are somewhat associated with fiber-enriched niches (Lee et al., 2008; Kuncharoen et al., 2018; Sakamoto et al., 2018; Abdugheni et al., 2022). This suggests that dietary fiber supplementation may foster microbial communities that influence host lipid metabolism, potentially affecting membrane composition, lipid signaling, and energy homeostasis. *Glutamicibacter protophormiae*, a potential steroid-degrading bacterium (Ghimire et al., 2024), correlated positively with APOB, which encodes apolipoprotein B involved in lipoprotein secretion, indicating a possible link between microbial metabolism and systemic lipid transport. Additional correlation of *Spirosoma, Eggerthella*, and *Blautia*, known fiber fermenters (Li et al., 2009; Malik et al., 2020; Pecyna et al., 2025), indicate mutualistic association that enhance fiber utilization in birds. Collectively, these associations highlight interconnected microbial-host pathways in response to dietary fiber, suggesting that the observed microbial shifts may contribute to different host physiological processes, though mechanistic validation is needed to establish causality.

This study demonstrates that different dietary fiber sources can modulate intestinal health during subclinical enteric infection through distinct but overlapping mechanisms. Combinations of insoluble and soluble fibers such as OH-WM, OH-SBP and SH-WM provided the most significant protection against challenge-induced intestinal pathology. The observed effects were fiber-specific, with OH generally exhibiting stronger anti-inflammatory properties while SH more robustly enhanced oxidative phosphorylation and metabolic pathways. These differences highlight the importance of considering fiber source when formulating diets for broilers at risk of enteric challenges.

While this study provides comprehensive insights into fiber-mediated modulation of intestinal health during subclinical enteric challenge, several limitations should be addressed in future research. First, the study examined a single time point post-challenge; a time-course analysis would provide valuable information on the dynamics of fiber-mediated protection. Second, direct measurement of microbial metabolites would complement the metagenomic functional pathways and provide more definitive evidence of altered microbial activity. Finally, validation of key transcriptomic findings at the protein level would strengthen the biological relevance of the observed gene expression changes.

In conclusion, dietary fiber supplementation represents a promising strategy for mitigating subclinical enteric challenge in broilers, with fiber combinations offering particular benefits through multiple complementary mechanisms. These findings contribute to our understanding of how dietary interventions can support intestinal health in the absence of in-feed antibiotics and highlight the importance of considering both fiber type and combinations when formulating diets for optimal intestinal health and disease resistance.

## CRediT authorship contribution statement

**R.W. Tabish:** Sample processing, formal analysis, writing – original draft preparation. **Y. Lin:** Conceptualization. **S.J. Rochell:** Conceptualization. **W.J. Pacheco:** Conceptualization. **M.A. Bailey:** Conceptualization. **W.A. Dozier III:** Conceptualization. **F.J. Hoerr:** Sample processing, formal analysis. **K. Robinson:** Conceptualization. **R. Hauck:** Conceptualization, supervision.

## Disclosures

The authors declare that they have no known competing financial interests or personal relationships that could have appeared to influence the work reported in this paper.

## Appendix Supplementary materials

Image, application 1

Image, application 2

Image, application 3

Image, application 4

## Footnotes

Supplementary material associated with this article can be found, in the online version, at doi:10.1016/j.psj.2026.107215.
